# Glances at the Hospitals

**Published:** 1900-10-27

**Authors:** 


### GLANCES AT THE HOSPITALS.

### THE NORTHAMPTON GENERAL INFIRMARY.

One hundred and fifty-six years have passed away since this now venerable institution issued its first annual report. Last year the ordinary income amounted to £7,730, and the ordinary expenditure to £7,316, the former being higher and the latter lower than the previous year. The statistics run from August 1st, 1898, to August 1st, 1899; but there does not seem to be any good reason for this arbitrary division of the year, and it would be preferable to begin on January 1st and finish on December 31st. There were 135 patients in the infirmary at the beginning of the year, and 1,408 were admitted during it either on letters or as accidents and emergencies. Of these in-patients 787 were discharged cured, 367 relieved, 164 were made out-patients, 34 were discharged for other reasons, and 105 died. One of the tables shows the cause and date of death of every patient who died during the year; and even the initials of the patients' names are given, so that they can be traced if necessary. Another table shows the nature and results of all the operations performed. We notice that there were six excisions of joints, and all were successful. There were 10 cases of strangulated hernia and 9 were successful, and 7 cases of operation for the radical cure of hernia, of which 6 were successful and 1 relieved.

### LAKESIDE HOSPITAL, CLEVELAND, OHIO.

On January 1st, 1899, this well-designed hospital contained 69 patients; 1,621 were admitted, making a total of 1,690 treated during the year. The *per capita* cost for all patients per diem has been $2 18c.; and as this sum has been obtained after deducting extraordinary expenses, it is very high indeed, or at least it would be considered very high in England. But it should be added that the hospital contains many private cases treated in private wards. Of the total number, 705 were discharged cured, 648 improved, 110 not improved, and 117 died. It is interesting to note the heterogeneous nativity of the patients. There were Assyrians, Austrians, Bavarians, Bohemians, Canadians (64), Chinese, Danes, English (69), French, Germans (128), Hungarians, Irish (79), Italians, Norwegians, Poles, Russians, Scotch (12), Swedes, Switzers, and Welsh. The average daily number resident in the hospital was 101 patients, and the daily average number of employés was 122. Of the latter 60 were nurses; but this number included 35 pupils. A training school for nurses is attached to the hospital. The medical and surgical tables are provided with seven columns, showing the numbers cured, improved, not improved, died and remaining. Curiously enough, the table of operations give no particulars as to recovery or death; but there is a carefully drawn-up table of the causes of all the deaths. Separate statistics are given of the gynæcological, the ophthalmological, the children's, the dermatological, and the pathological divisions. Like all American public documents, the report is very well got up, and contains many illustrations.

